# One-Pot Exfoliation of Graphite and Synthesis of Nanographene/Dimesitylporphyrin Hybrids

**DOI:** 10.3390/ijms160510704

**Published:** 2015-05-12

**Authors:** M. Mar Bernal, Emilio M. Pérez

**Affiliations:** IMDEA Nanociencia, C/Faraday 9, Ciudad Universitaria de Cantoblanco, Madrid 28049, Spain; E-Mail: mar.bernal@imdea.org

**Keywords:** graphene, porphyrin, π–π stacking interactions, van der Waals forces

## Abstract

A simple one-pot process to exfoliate graphite and synthesize nanographene-dimesitylporphyrin hybrids has been developed. Despite the bulky mesityl groups, which are expected to hinder the efficient π–π stacking between the porphyrin core and graphene, the liquid-phase exfoliation of graphite is significantly favored by the presence of the porphyrins. Metallation of the porphyrin further enhances this effect. The resulting graphene/porphyrin hybrids were characterized by spectroscopy (UV-visible, fluorescence, and Raman) and microscopy (STEM, scanning transmission electron microscopy).

## 1. Introduction

Graphene, one-atom thick sheet of *sp*^2^ hybridized carbon atoms arranged in a honeycomb lattice, has emerged as an attractive 2D nanomaterial owing to its outstanding physicochemical properties [1,2,3,4,5,6,7,8]. Several synthetic routes to produce graphene have been developed in the last few years [9,10], but the most straightforward method of production is still the exfoliation from graphite, a natural and abundant source of carbon. In graphite, graphene flakes are stacked due to large van der Waals and π–π interactions contributing to the high-thermodynamic stability of graphite. To produce graphene, it is necessary to disrupt these non-covalent forces without sacrificing the *sp*^2^ conjugated network responsible of its unique electronic properties [11,12]. Among the different strategies to deal with this problem [13,14], micromechanical cleavage [4,15] and liquid-phase exfoliation of graphite [16,17] are the most widely used. Although the good-quality and large size of the sheets generated by micromechanical exfoliation of graphite are a definite advantage, its low yield limits its application to fundamental research. On the other hand, exfoliation of graphite through ultrasonication in a solvent of adequate surface tension is a cost-effective process and yields bulk amounts of few layer graphene (FLG). Once exfoliated, functionalization of graphene is required for many applications, from composite materials to electronic devices [18,19]. In this regard, aromatic molecules have proven to be effective adjuvants in the exfoliation of graphite, producing novel hybrids with exciting optoelectronic properties in a single step [20,21,22,23,24,25].

Porphyrins are electron-rich organic molecules with unique optical and electronic properties, which can be tuned through molecular engineering. For example, porphyrin derivatives have been used in photodynamic therapy, catalysis, photovoltaic devices, and molecular electronics [26,27,28]. Porphyrins have been widely used to decorate carbon materials such as fullerenes and carbon nanotubes in a supramolecular fashion, because of their strong π–π stacking interactions with them. Moreover, the electron-donor character of porphyrins can promote photoinduced electron transfer processes with carbon-based materials for the development of optoelectronic devices [29,30,31,32,33]. To enhance π–π interactions between graphene and the porphyrin core, porphyrins bearing either simple alkyl or benzyl substituents in the meso positions have been employed [33,34]. However, most porphyrin derivatives bear bulky aromatic substituents in the meso positions, which enhance solubility.

Here, we describe the preparation of graphene-dimesitylporphyrin hybrids by direct exfoliation of graphite in a solution of the porphyrins. This methodology provides access to the nanohybrids in one step without further functionalization or purification steps. Addition of the porphyrins significantly enhances the yield of exfoliation of graphite, in spite of the presence of the bulky mesityl groups. The resulting porphyrin-FLG hybrids are stable in suspension after several months without any sign of precipitation, and could find applications in graphene-based optoelectronic devices.

## 2. Results and Discussion

**Figure 1:**
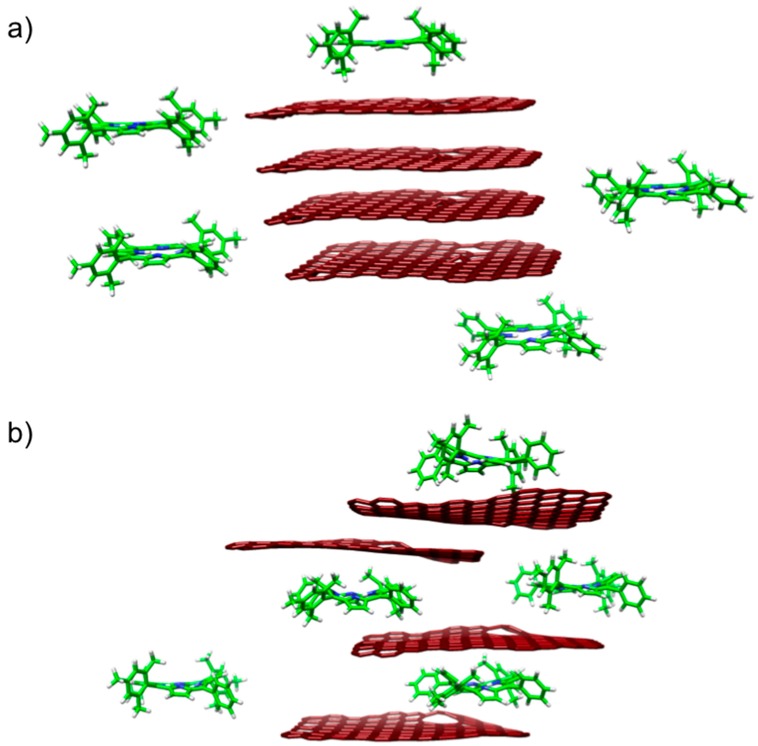
Cartoon representation of the exfoliation mechanism to produce few layer graphene (FLG)/porphyrin hybrids: (**a**) Addition of graphite to porhyrin solution; and (**b**) Porphyrin molecules exfoliate graphene sheet to produce FLG/porphyrin hybrids.

The yield and quality of exfoliated FLG dispersed in organic solvents depends on different factors, such as solvent, sonication time and energy, initial concentration of graphite, centrifugation rates, *etc.* [35]. In this study, we use an initial concentration of graphite of 1 mg·mL^−1^, which was directly exfoliated in solutions of the porphyrins in NMP without the addition of surfactants or ions (Figure 1). In particular, we have used 5,15-dimesityl-10,20-diphenylporphyrin (DMPP) and its metallated derivative Zn-5,15-dimesityl-10,20-diphenylporphyrin (ZnDMPP). It has been proven before that planarity is not a prerequisite for the efficient interaction of conjugated molecules and FLG [36]. The main aim of our study was to investigate if bulky substituents in the meso positions of the porphyrins would be detrimental for their interaction with graphene. 

**Figure 2:**
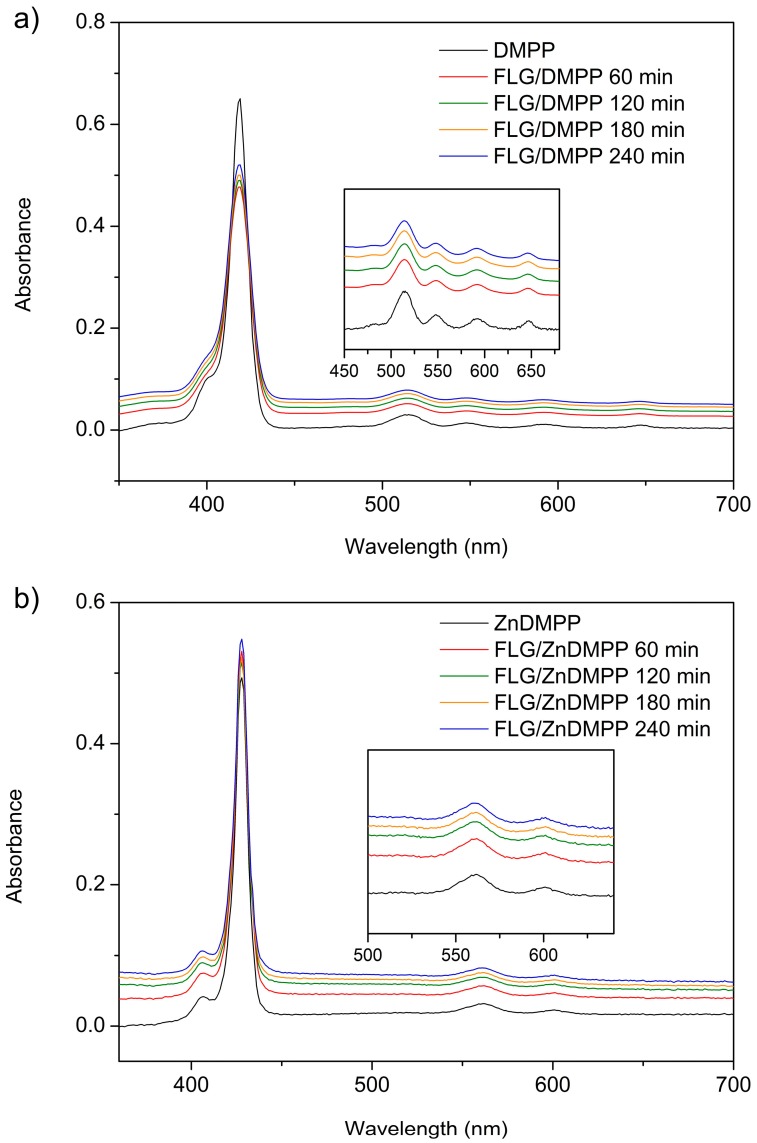
UV-vis absorption spectra of FLG/porphyrin hybrids recorded at different exfoliation times of graphene (**a**) FLG/DMPP; and (**b**) FLG/ZnDMPP. Insets show a magnification of the Q-bands in each case, which show similar changes to those observed for the Soret band.

The interplay between FLG and the porphyrin molecules during the exfoliation process was first studied by UV-vis spectroscopy. The absorbance changes recorded for the dispersions of FLG/porphyrin hybrids at different exfoliation times are shown in Figure 2. DMPP exhibits a strong Soret band at 418 nm and four Q-bands located between 500 and 650 nm (Figure 2a). On the other hand, the absorption spectrum of ZnDMPP shows a redshift of the Soret band to 427 nm, while the number of Q-bands decreases to two due to the coordination with Zn^2+^ (Figure 2b). Upon interaction with graphene, the most obvious change is the decrease in the relative intensity of the Soret band, which has been previously observed for similar systems [33] and is an indication of the porphyrin–graphene interaction. In particular, we observed a decrease in the intensity of the Soret band of up to 30% for DMPP and 35% for ZnDMPP, which points to a more effective interaction in the case of ZnDMPP. Other than that, there are no obvious spectral shifts during the exfoliation process.

**Figure 3:**
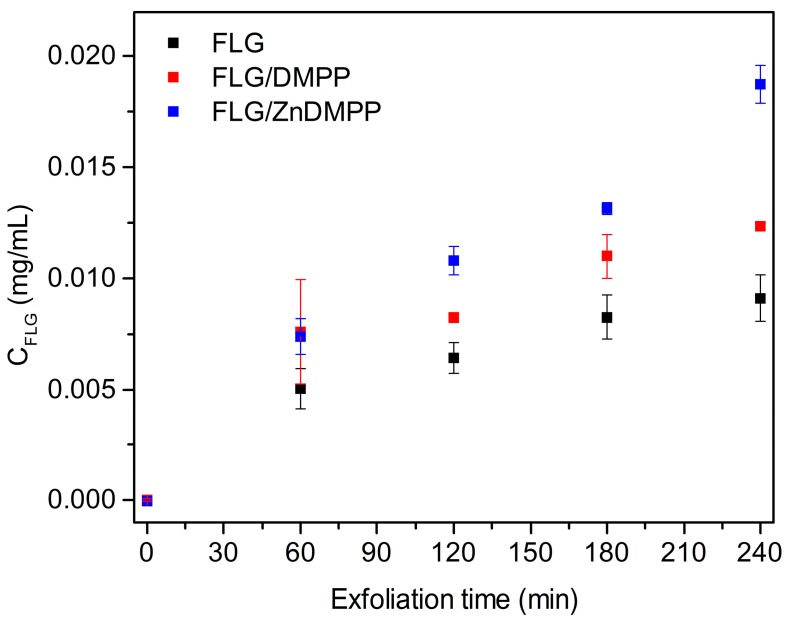
Concentration of FLG (*C*_FLG_) *vs.* exfoliation time obtained in pure 1-methyl-2-pyrrolidone (NMP) (black squares) and in the different porphyrin solutions. Each data point represents the mean of three separate experiments and error bars are standard deviations.

The amount of graphene dispersed as function of exfoliation time was determined using the absorption coefficient α = 3620 mL·mg^−1^·m^−1^ at 670 nm (Figure 3) [33,37]. At this wavelength porphyrin molecules do not show any absorption (Figure 2, black traces) so the absorbance measured is related exclusively to the concentration of graphene in the dispersions. For comparison purposes, graphene was exfoliated in pure NMP under the same conditions used for FLG/porphyrin hybrids. Starting from the same concentration of graphite (1 mg·mL^−1^) our results clearly demonstrate that the presence of porphyrins enhances the incorporation of graphene to the dispersions. Quantitatively, for a sonication time of 4 h we obtained 0.008 ± 0.001 mg·mL^−1^ of FLG in the NMP sample, compared to 0.012 ± 0.002 mg·mL^−1^ in the FLG/DMPP sample and 0.019 ± 0.009 mg·mL^−1^ in the FLG/ZnDMPP sample, that is, an increase in the yield of FLG of *ca.* 50% and 140%, respectively.

**Figure 4:**
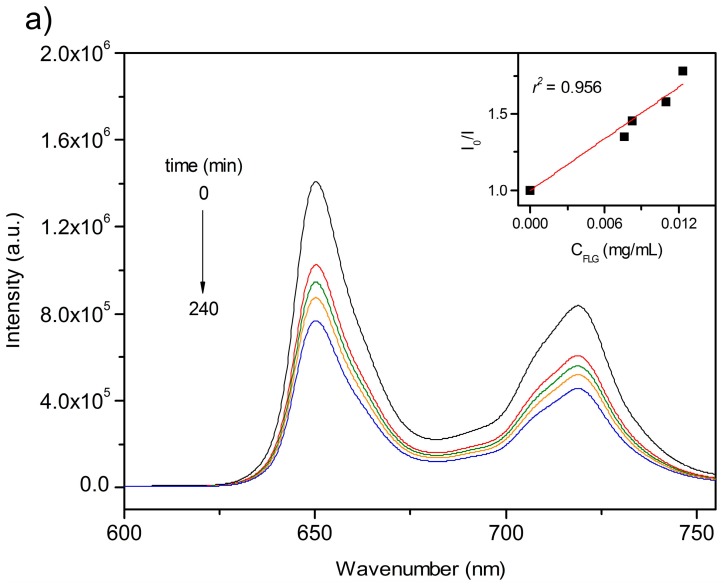

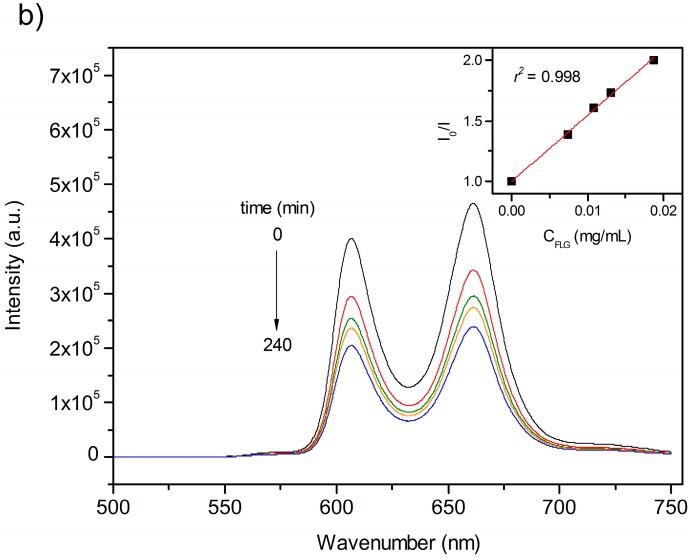
Fluorescence emission spectra of nanographene/porphyrin hybrids recorded at different exfoliation times of graphene (**a**) FLG/DMPP (λ_exc_ = 418 nm); and (**b**) FLG/ZnDMPP (λ_exc_ = 427 nm). The insets are the Stern-Volmer plots of *I*_0_/*I vs.* *C*_FLG_ for FLG/DMPP (recorded at λ = 650 nm) and FLG/ZnDMPP (recorded at λ = 606 nm).

The changes in fluorescence intensity upon increasing graphene concentration are displayed in Figure 4. No spectral shifts were observed for either DMPP or ZnDMPP upon interaction with graphene. However, the emission intensity decreases gradually as the concentration of graphene increases.

The fluorescence quenching can be quantitatively evaluated by the Stern-Volmer (SV) equation (Equation (1)):

(1) $$I_{0}/I=1+K_{\text{G}}C_{\text{G}}$$

where *C*_G_ represents the concentration of graphene (quencher), *I*_0_ and *I* are the fluorescence intensities of porphyrins in the absence and presence of graphene respectively, and *K*_G_ is the quenching constant of fluorescence [38,39]. The *K*_G_ obtained for FLG/DMPP and FLG/ZnDMPP from the SV formula were 53.2 and 59.5 mL·mg^−1^, respectively, in line with a more efficient FLG/porphyrin interaction for the ZnDMPP hybrid, in accordance with the absorbance data.

**Figure 5:**
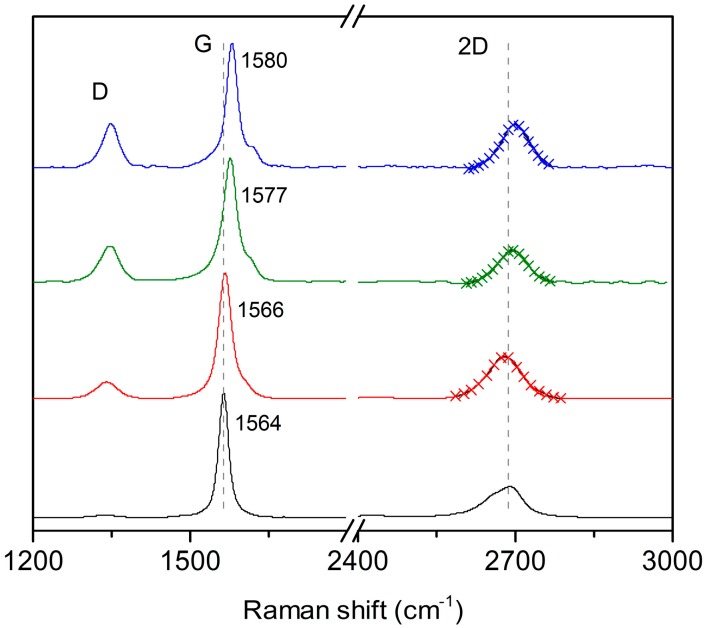
Raman spectra (mean of five separate measurements) of graphite (black) and FLG in NMP (red), FLG/DMPP (green) and FLG/ZnDMPP (blue) after exfoliation during 240 min. Cross symbols represent the single Lorentzian fit of the 2D band.

Finally, the interaction between porphyrins and graphene was also investigated through Raman spectroscopy (Figure 5). As expected, we could not record the Raman spectra of the porphyrins due to their strong fluorescence, however this is not the case for the FLG/porphyrin hybrids due to graphene-induced fluorescence quenching (see Figure 4) [40]. The Raman spectra of graphene exfoliated in pure NMP and FLG/porphyrin hybrids after 240 min show a noticeable change in the 2D band with respect to graphite, with all three samples showing a much more symmetric shape indicative of FLG sheets. In fact, a single Lorentzian fit of the 2D band is sufficient in each case (Figure 5). The FWHM (full-width at half maximum) and the ratio of the 2D/G band intensities are reported in Table 1. FWHM of the 2D band decreases with the number of layers in few-layer graphene, while the 2D/G ratio is sensitive to both the number of layers, increasing with decreasing number of layers and reaching up to three in single-layer graphene, and strongly sensitive to electron doping, decreasing on interaction with electron-donor and -acceptor molecules [41,42]. Interestingly, both parameters decrease for FLG/porphyrin hybrids compared with graphene exfoliated in NMP. As expected, the FWHM decreases significantly from graphite (89 cm^−1^) to NMP-exfoliated graphene (86 cm^−1^). Significantly, this effect is more pronounced in FLG/DMPP (78 cm^−1^) and even more in FLG/ZnMPP (75 cm^−1^). These results indicate that not only the yield, but also the efficiency of the exfoliation process is enhanced upon addition of the porphyrins. Meanwhile, the 2D band intensity diminishes due to the electron doping of graphene by porphyrin molecules, as corroborated by the significant shifts in the frequency of the G band and the decrease of the 2D/G ratio (Figure 5). On the other hand, a shoulder on the G band, referred as G’ band, appears in FLG, related to defects [41], which upshifts following the same trend as the G band. Furthermore, the intensity of the D band, usually related to the presence of in-plane defects or graphene edges, increases for the FLG/porphyrin hybrids, which is also in agreement with smaller graphene flakes.

**Table 1:** Full-width at half maximum (FWHM) and 2D/G ratio of graphite, FLG and FLG/porphyrin hybrids.

Sample	FWHM (cm^−1^)	2D/G
Graphite	89	0.25
FLG	86	0.82
FLG/DMPP	78	0.74
FLG/ZnDMPP	75	0.66

Therefore, the changes observed in UV-vis, fluorescence, and Raman are consistent with an enhancement of both the yield and efficiency of the exfoliation process in the presence of DMPP and ZnDMPP when compared to pure NMP.

**Figure 6:**
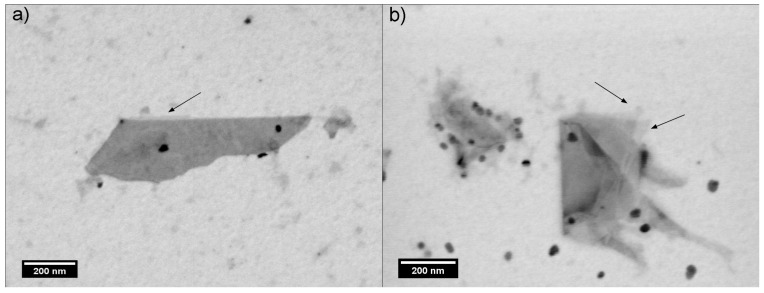
Scanning transmission electron microscopy (STEM) images of FLG/porphyrin hybrids: (**a**) FLG/DMPP and (**b**) FLG/ZnDMPP. The areas marked with arrows correspond to single-layer graphene.

The morphologies of FLG/porphyrin hybrids were investigated by scanning transmission electron microscopy (STEM). Figure 6 shows folded graphene layers, which show regular and flat morphologies. In particular, by looking at the folded edges, single-(marked with arrows) and few-layer sheets are observed, confirming the successful exfoliation of graphite. Furthermore, some of the flakes are covered with nanocrystals of porphyrin molecules, which are aggregated during the preparation of the TEM grid [33].

## 3. Experimental Section

### 3.1. General

Graphite powder (<45 μm, 99.99%) and all reagents were purchased from Aldrich (Saint Louis, MO, USA). Solvents were purchased from commercial suppliers and used as received. UV-visible spectra of three different samples of each graphene solution were measured by using a Cary 50 UV-visible spectrophotometer (Varian, Bergen op Zoom, The Netherlands) in quartz cuvettes with a pathlength of 1 cm. Fluorescence experiments were carried out at least three times for each sample. Emission spectra were recorded on a NanoLog 4 HORIBA (Jobin Yvon, Cedex, France). Raman spectra were acquired with a Bruker Senterra confocal Raman microscopy instrument (Bruker Optiks, Ettlingen, Germany), at excitation laser wavelength of 532 nm. The laser power at the sample was 10 mW, and the data acquisition time was 20 s. The sample preparation involved drop casting of the solutions on a Si substrate with a 300 nm oxide layer. STEM images were obtained with an Auriga Carl ZEISS operating at 25 kV (Carl Zeiss Microscopy GmbH, Jena, Germany). The FLG/porphyrin hybrids were deposited by drop casting of the dispersions onto holey carbon mesh grid. 

### 3.2. Preparation of Graphene/Porphyrin Hybrid Materials

Graphene dispersions were prepared via a liquid-phase exfoliation technique by adding graphite powder to 1-methyl-2-pyrrolidone (NMP) at a concentration of 1 mg·mL^−1^. The solutions were sonicated (Fisherbrand FB15051 bath sonicator, maximum operating power 320 W, Fisher Scientific, Loughborough, UK) for various periods from 60 to 240 min. In order to exclude the effect of temperature and to avoid the degradation of the solvent due to an increase of the bath water during sonication, a cooling system was connected to the bath sonicator performing the experiments at 20 °C. At different times during this period, aliquots of the suspensions were centrifuged at 3000 rpm for 30 min (Hettich Zentrifugen Universal 320, Andreas Hettich GmbH & Co. KG, Tuttlingen, Germany). After that, the supernatant was carefully collected for further characterization. For FLG/porphyrin hybrids, the same procedure was followed but graphite was added to solutions of 5,15-dimesityl-10,20-diphenylporphyrin (0.01 mg·mL^−1^ in NMP) and Zn-5,15-dimesityl-10,20-diphenylporphyrin (0.01 mg·mL^−1^). In this case, the supernatant of FLG/porphyrin hybrids was directly measured as obtained.

## 4. Conclusions

The one-pot exfoliation of graphite and formation of FLG/DMPP hybrids has been demonstrated. The presence of the two bulky mesityl groups in the porphyrins does not impede their interaction with FLG. In fact, UV-vis measurements show that the presence of the porphyrins enhances the concentration of exfoliated graphene by up to 140%. We have also observed a very significant effect of the metal core of the porphyrin on the interaction with the surface of graphene. Our results show that the decrease in the efficiency of π–π interactions between the aromatic core of porphyrins and FLG can be compensated for by adequate CH–π and van der Waals interactions. This expands the range of porphyrins that can be used to synthesize FLG/porphyrin hybrids to include those with bulky substituents in the meso positions, which in turn should facilitate their use in electronic devices.

## References

[1] Balandin A.A., Ghosh S., Bao W., Calizo I., Teweldebrhan D., Miao F., Lau C.N. (2008). Superior thermal conductivity of single-layer graphene. Nano Lett..

[2] Stoller M.D., Park S., Zhu Y., An J., Ruoff R.S. (2008). Graphene-based ultracapacitors. Nano Lett..

[3] Lee C., Wei X., Kysar J.W., Hone J. (2008). Measurement of the elastic properties and intrinsic strength of monolayer graphene. Science.

[4] Novoselov K.S., Geim A.K., Morozov S.V., Jiang D., Zhang Y., Dubonos S.V., Grigorieva I.V., Firsov A.A. (2004). Electric field effect in atomically thin carbon films. Science.

[5] Geim A.K., Novoselov K.S. (2007). The rise of graphene. Nat. Mater..

[6] Du X., Skachko I., Barker A., Andrei E.Y. (2008). Approaching ballistic transport in suspended graphene. Nat. Nanotechnol..

[7] Zhang Y., Tan Y.-W., Stormer H.L., Kim P. (2005). Experimental observation of the quantum hall effect and berry’s phase in graphene. Nature.

[8] Novoselov K.S., Geim A.K., Morozov S.V., Jiang D., Katsnelson M.I., Grigorieva I.V., Dubonos S.V., Firsov A.A. (2005). Two-dimensional gas of massless dirac fermions in graphene. Nature.

[9] Zhang Y., Zhang L., Zhou C. (2013). Review of chemical vapor deposition of graphene and related applications. Acc. Chem. Res..

[10] Chen L., Hernández Y., Feng X., Müllen K. (2012). From nanographene and graphene nanoribbons to graphene sheets: Chemical synthesis. Angew. Chem. Int. Ed..

[11] Eigler S., Hirsch A. (2014). Chemistry with graphene and graphene oxide—Challenges for synthetic chemists. Angew. Chem. Int. Ed..

[12] Pénicaud A., Drummond C. (2012). Deconstructing graphite: Graphenide solutions. Acc. Chem. Res..

[13] Vázquez E., Giacalone F., Prato M. (2014). Non-conventional methods and media for the activation and manipulation of carbon nanoforms. Chem. Soc. Rev..

[14] Quintana M., Vázquez E., Prato M. (2013). Organic functionalization of graphene in dispersions. Acc. Chem. Res..

[15] Geim A.K. (2009). Graphene: Status and prospects. Science.

[16] Yang H., Hernández Y., Schlierf A., Felten A., Eckmann A., Johal S., Louette P., Pireaux J.J., Feng X., Müllen K. (2013). A simple method for graphene production based on exfoliation of graphite in water using 1-pyrenesulfonic acid sodium salt. Carbon.

[17] Coleman J.N. (2012). Liquid exfoliation of defect-free graphene. Acc. Chem. Res..

[18] Sreeprasad T.S., Berry V. (2013). How do the electrical properties of graphene change with its functionalization?. Small.

[19] Roppolo I., Chiappone A., Bejtka K., Celasco E., Chiodoni A., Giorgis F., Sangermano M., Porro S. (2014). A powerful tool for graphene functionalization: Benzophenone mediated UV-grafting. Carbon.

[20] Zhang M., Parajuli R.R., Mastrogiovanni D., Dai B., Lo P., Cheung W., Brukh R., Chiu P.L., Zhou T., Liu Z. (2010). Production of graphene sheets by direct dispersion with aromatic healing agents. Small.

[21] Zhang F., Chen X., Boulos R.A., Md Yasin F., Lu H., Raston C., Zhang H. (2013). Pyrene-conjugated hyaluronan facilitated exfoliation and stabilisation of low dimensional nanomaterials in water. Chem. Commun..

[22] Wang W., Zhang Y., Wang Y.-B. (2014). Noncovalent π···π interaction between graphene and aromatic molecule: Structure, energy, and nature. J. Chem. Phys..

[23] Ghosh A., Rao K.V., George S.J., Rao C.N.R. (2010). Noncovalent functionalization, exfoliation, and solubilization of graphene in water by employing a fluorescent coronene carboxylate. Chem. Eur. J..

[24] Costa R.D., Malig J., Brenner W., Jux N., Guldi D.M. (2013). Electron accepting porphycenes on graphene. Adv. Mater..

[25] Luo B., Liu S., Zhi L. (2012). Chemical approaches toward graphene-based nanomaterials and their applications in energy-related areas. Small.

[26] Ethirajan M., Chen Y., Joshi P., Pandey R.K. (2011). The role of porphyrin chemistry in tumor imaging and photodynamic therapy. Chem. Soc. Rev..

[27] Martínez-Díaz M.V., de la Torre G., Torres T. (2010). Lighting porphyrins and phthalocyanines for molecular photovoltaics. Chem. Commun..

[28] Benniston A.C. (2007). Porphyrin linked poly(pyridyl)-based conjugates as artificial photosynthetic reaction centre models. Phys. Chem. Chem. Phys..

[29] Guo Z., Du F., Ren D., Chen Y., Zheng J., Liu Z., Tian J. (2006). Covalently porphyrin-functionalized single-walled carbon nanotubes: A novel photoactive and optical limiting donor-acceptor nanohybrid. J. Mater. Chem..

[30] Xu H., Wu P., Liao C., Lv C., Gu Z. (2014). Controlling the morphology and optoelectronic properties of graphene hybrid materials by porphyrin interactions. Chem. Commun..

[31] Aly S.M., Parida M.R., Alarousu E., Mohammed O.F. (2014). Ultrafast electron injection at the cationic porphyrin-graphene interface assisted by molecular flattening. Chem. Commun..

[32] Malig J., Stephenson A.W.I., Wagner P., Wallace G.G., Officer D.L., Guldi D.M. (2012). Direct exfoliation of graphite with a porphyrin—Creating functionalizable nanographene hybrids. Chem. Commun..

[33] Kiessling D., Costa R.D., Katsukis G., Malig J., Lodermeyer F., Feihl S., Roth A., Wibmer L., Kehrer M., Volland M. (2013). Novel nanographene/porphyrin hybrids—Preparation, characterization, and application in solar energy conversion schemes. Chem. Sci..

[34] Geng J., Kong B.-S., Yang S.B., Jung H.-T. (2010). Preparation of graphene relying on porphyrin exfoliation of graphite. Chem. Commun..

[35] Hernández Y., Nicolosi V., Lotya M., Blighe F.M., Sun Z., De S., McGovern I.T., Holland B., Byrne M., Gun’Ko Y.K. (2008). High-yield production of graphene by liquid-phase exfoliation of graphite. Nat. Nanotechnol..

[36] Brunetti F.G., Isla H., Aragó J., Ortí E., Pérez E.M., Martín N. (2013). Exploiting multivalent nanoparticles for the supramolecular functionalization of graphene with a nonplanar recognition motif. Chem. Eur. J..

[37] Khan U., O’Neill A., Lotya M., De S., Coleman J.N. (2010). High-concentration solvent exfoliation of graphene. Small.

[38] Huang T., Murray R.W. (2002). Quenching of [Ru(bpy)_3_]^2+^ fluorescence by binding to au nanoparticles. Langmuir.

[39] Zhu M., Li Z., Xiao B., Lu Y., Du Y., Yang P., Wang X. (2013). Surfactant assistance in improvement of photocatalytic hydrogen production with the porphyrin noncovalently functionalized graphene nanocomposite. ACS Appl. Mater. Interfaces.

[40] Xie L., Ling X., Fang Y., Zhang J., Liu Z. (2009). Graphene as a substrate to suppress fluorescence in resonance raman spectroscopy. J. Am. Chem. Soc..

[41] Rao C.N.R., Biswas K., Subrahmanyam K.S., Govindaraj A. (2009). Graphene, the new nanocarbon. J. Mater. Chem..

[42] Das B., Voggu R., Rout C.S., Rao C.N.R. (2008). Changes in the electronic structure and properties of graphene induced by molecular charge-transfer. Chem. Commun..

